# Supplementary material for: Lignosus rhinocerotis Cooke Ryvarden ameliorates airway inflammation, mucus hypersecretion and airway hyperresponsiveness in a murine model of asthma
Source: PLoS One. 2021 Mar 30;16(3):e0249091. doi: 10.1371/journal.pone.0249091 (PMC8009377; doi:10.1371/journal.pone.0249091)
Supplement: S1 File — (DOCX) [file pone.0249091.s002.docx]

**S1 File**

***L. rhinocerotis* extraction**

For preparation of hot water extract, the dried crude powder was extracted using hot water percolation in a sohxlet. Firstly, 50 g *L. rhinocerotis* sclerotium was inserted into an extraction thimble (22 mm internal diameter and 90 mm external length) and 600 ml of purified distilled water was used as a solvent. Following extraction, the extract was filtered using a filter paper before being concentrated with a rotary evaporator followed by freeze drying (into a lyophilized powder form). Extraction of 50 g mushroom powder yielded approximately 5 g of *L. rhinocerotis* extract (LRE).

**Ovalbumin (OVA)-induced rat model of asthma**

**Sensitization, challenge and treatment**

Male Sprague Dawley rats, age between 6-8 weeks (200 -300 g) were used in this experiment. The animals were acclimatized for one week prior to the experiment. The animals were randomised into six groups (n=5 /group): 1) normal group 2) OVA, sensitised and challenged with 1% OVA 3) LRE125, sensitised and challenged with OVA; treated with oral LRE (125 mg/kg) (4) LRE250, sensitised and challenged with OVA; treated with oral LRE (250 mg/kg) (5) LRE500, sensitised and challenged with OVA; treated with oral LRE (500 mg/kg) (4) Dex, LRE125, sensitised and challenged with OVA; treated with dexamethasone (3 mg/kg). The challenge and treatment periods were carried out for seven days. On day 23, the rats were challenged by an aerosol of 1% OVA for 20 min/day for 7 days. The animals were placed in a restrainer tube and were given aerosolized 1% OVA solution via an ultrasonic nebulizer (Mabist mist, USA) (Figure A). The rats were administered with oral injection of respective dosage of LRE after each challenge for 7 days. The animals were sacrificed within 24 hours after the last challenge on day 29. The optimal dosage for the subsequent study was then selected based on this study.


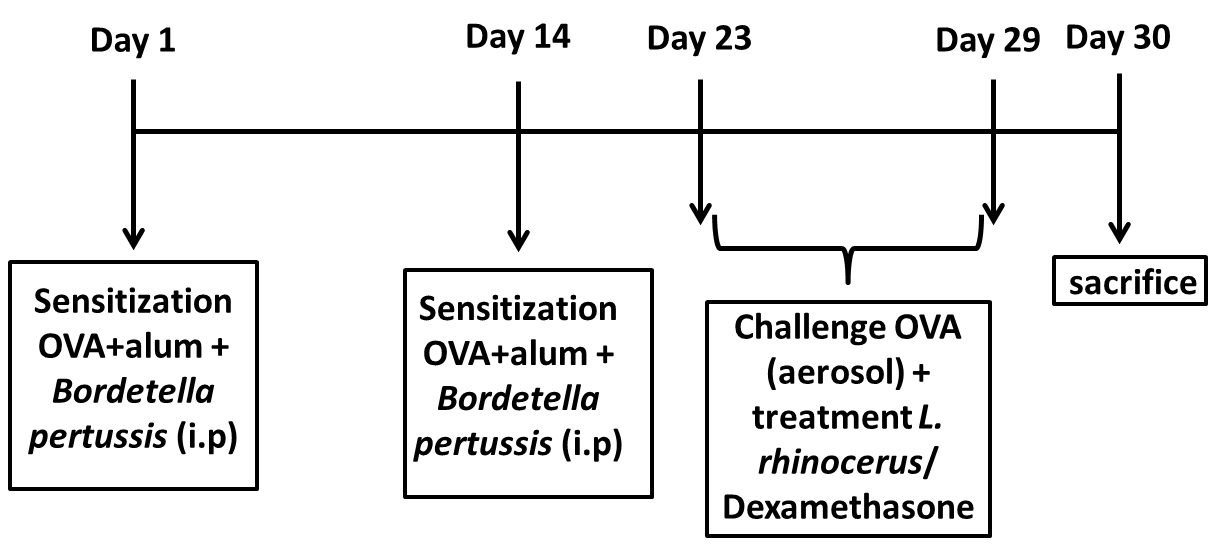


**Figure A.** Experimental protocol for establishment of OVA-induced airway inflammation rodent model and treatment.

**Sacrifice, blood and bronchoalveolar lavage fluid collections and lung retrieval**

Sprague Dawley rats were anesthetized with ketamine and xylazine at 100 mg/kg and 10 mg/kg per body weight respectively. Blood (5 ml) was collected from all rats by cardiac puncture. Serum was obtained by centrifuging the blood at 500 g for 10 minutes and then stored at -70°C until used. BALF was obtained from the rats’ trachea using an endotracheal tube by instillation and aspiration with 3 ml PBS solution. BALF was then transferred into a centrifuge tube, centrifuged at 500 g for 10 minutes, and the supernatant was collected and stored at -70°C for cytokine analysis. BALF pellet was used for eosinophils and total cell counts. The lungs were removed, rinsed with PBS, weighed and fixed in 10% formalin overnight for histology analysis.

**Measurement of total IgE in serum**

Total immunoglobulin E (IgE) was measured by specific rat ELISA kit according to the manufacturer's instructions. Serum (100 μl) was added into the pre-coated well of anti-IgE antibodies and incubated for 1 hour at room temperature. The contents of the wells were then aspirated. This was followed by the addition of 100 μl of enzyme-antibody conjugate before the reaction was incubated for 1 hour at room temperature for 60 minutes in the dark. The plate was then washed for four times (2 minutes each). Finally, the enzyme bound to the immunosorbent was assayed by the addition of 3,3',5,5'-Tetramethylbenzidine (TMB). The stop solution was then added. IgE level in each sample was measured using optical density at 450 nm followed by a calculation based on a standard curve that was generated using a recombinant IgE.

**Measurement of T helper 2 (Th2) cytokines in BALF**

An ELISA was performed according to the manufacturer's instructions. Interleukin (IL)-4, IL-5 and IL-13 in BALF were measured using specific rat IL-4, IL-5 and IL-13 ELISA kits. BALF supernatant (100 μl) was added in the pre-coated well of specific antibody and was incubated for 2 hours at 37°C. This was followed by the addition of 100 μl of biotin-antibody and the reaction incubated for 1 hour at 37°C. The wells were aspirated and washed for three times (2 minutes each). Then, 100 μl of HRP-avidin was added into each well followed by incubation for 1 hour in the dark. After that, the reaction was washed for four times as above, followed by the addition of 90 μl of TMB substrate. The samples were incubated for 30 minutes at 37°C followed by the stop solution. The bound enzyme that was proportional to the respective cytokine concentration was measured at absorbance 450 nm. Cytokine’s concentrations were calculated from standard curves generated using similar type of recombinant interleukins.

**Histopathological analysis of the lungs**

After sacrifice, the rat’s lungs were fixed by immersing in 10% formalin overnight. The lungs were sliced into sections and placed in a tissue cassette overnight for tissue processing which involved dehydration, clearing and paraffin wax infiltration. Fixed specimens were dehydrated in 70%, 95%, 95%, 100% (1), 100% (2) and 100% (3) ethanol for 1.5 hour respectively. For the clearing process, the tissue cassettes were fixed with 50:50 solution (100% ethanol: xylene) for 1 hour, followed by fixation in xylene (1) and xylene (2) for 1 hour respectively. In infiltration (embedding), the paraffin wax was used at 56-57°C for 1 hour. After completion, the lungs tissues, were embedded with paraffin before being allowed to cool. Paraffin embedded tissue sections were fixed (4 µm) on polysine coated slides and were dried overnight at 37°C.

**Haematoxylin and eosin staining**

The lungs tissue sections were deparaffinized with two changes of xylene for 10 minutes each. The tissues were then rehydrated in two changes of 100% alcohol for 5 minutes each. Following fixation in 95% alcohol and 70% alcohol for 2 minutes respectively, the slides were washed briefly in distilled water. Then, the slides were stained in Harris hematoxylin solution for 8 minutes before washing in a running tap water for 5 minutes. Next, the slides were differentiated into a 1% acid alcohol for 30 seconds before washing for 1 minute. For bluing step, the slides were fixed in 0.2% ammonia water for 10 seconds. The slides were washed for 5 minutes and rinsed in 95% alcohol, counterstained in Eosin solution for 1 minute then dehydrated through alcohol series of 95% and 2 changes of 100% alcohol, 5 minutes each. Finally, the slides were cleared by two changes of xylene for 5 minutes each. The tissues were subsequently mounted and cover-slipped with di-n-butyl phthalate in xylene (DPX mount). Eosinophil cell infiltration into peribronchial/perivascular areas was evaluated and graded according to the following criteria adapted from Lee *et al*. (2007). The whole lung sections were then assigned with a scoring system between 0 and 4 where;

0: no cells

1: a few cells

2: a ring of cells 1 cell layer deep

3: a ring of cells 2–4 cells deep

4: a ring of cells > 4 cells deep

For each rat, randomly distributed airway sections in the lung were analysed and their mean scores were calculated. The inflammation was evaluated by a double-blind scoring method. Quantitative analysis of lung inflammation score was performed by using a Mirax Image viewer. The cell infiltration intensity of inflammation at peribronchial and perivascular regions was scored as the approximate number of cells layers around the vessels or the bronchioles. Most severe region of infiltration was viewed as the representative section since each tissue section has infiltration intensity that varies for each vessel and airway (Jeong *et al.*, 2010).

**Periodic acid–Schiff (PAS) staining**

The lung tissue sections were deparaffinized with two changes of xylene of, 10 minutes each. The tissues were then re-hydrated with two changes of 100% alcohol for 5 minutes each. Following fixation in 95% alcohol and 70% alcohol for 2 minutes respectively, slides were washed briefly in distilled water. The slides were stained in PAS for 5 minutes before a washing step in running water for 5 minutes. Slides were counterstained in Gill III Haematoxylin solution for 90 seconds. The slides were rinsed again in water and then dehydrated through alcohol series of 95% and two changes of 100% alcohol for 5 minutes each. Finally, the slides were cleared at two changes of xylene, 5 minutes each. The tissues were subsequently mounted and cover-slipped with DPX mount. Goblet-cell hyperplasia in airway epithelium was quantified based on a five-point system (Lee *et al.*, 2010) where;

0: no goblet cells

1: <25% of epithelium

2: 25–50% of epithelium

3: 51–75% of epithelium

4: >76% of epithelium

Randomly distributed airways were analysed, and the average scores were quantified for mucus production using Mirax Image viewer.

9

inflammation [20]. Goblet-cell hyperplasia in airway epithelium was calculated based on a

five-point system where “0”: no goblet cells, “1”: <25% of epithelium, “2”: 25–50% of

epithelium, “3”: 51–75% of epithelium and “4” >76% of epithelium [21]. The mean scores

for mucus production were similarly calculated by means of a Mirax Image viewer (Carl

Zeiss, Germany).

Cell subsets analysis by flow cytometry

**Cell subsets analysis by flow cytometry**

BALF pellet of 1×10^6^ total cells were stained with antibodies namely CD4^+^ FITC, CD25^+^ PE and Foxp3^+^ APC (1 μg/ml concentration each) for detection of regulatory T cells (Jin *et al.*, 2013). The cells were stained for 30 minutes at 4°C in the dark. Next, the cells were fixed and permeabilized using fixation/permeabilization solution for 30 minutes and were subsequently stained with anti-rat Foxp3 PE. The cells were resuspended in 500 μl PBS and were analysed by an Accuri flow cytometry.

For eosinophils detection, the cells were stained with CD3^+^ PE (to stain T cells), RP-1^+^ PE (to stain neutrophils) and HIS48^+^ FITC (to stain eosinophils) (Werner-Klein *et al.*, 2008) at 1 μg/ml concentration each. The cells were resuspended in 500 μl PBS and were analysed by Accuri flow cytometry. A single tube was used with antibodies combination. All antibody concentrations for staining were based on the data supplied by the manufacturers following the initial optimizations. Background fluorescence was assessed by the appropriate isotype and the relevant fluorochrome control monoclonal antibody to define the percentage of positive cells.

**Allergy and asthma pathway-related genes array analysis**

**RNA extraction**

RNA samples of high quality were ensured by maintaining the appropriate working environment, laboratory apparatus such as electrophoresis chamber, pipettors and other non-disposable plasticwares and glass wares sterilized with RNase Zap^®^ to avoid contamination by RNases. The pipette tips and microcentrifuge tubes were sterilized to be RNase-free. A working solution of 70% (v/v) alcohol was prepared using DEPC-treated water for an RNase-free environment. RNA was isolated from lung tissues harvested from all group of animals which were sacrificed and stabilized in RNAlater solution at -80ºC. RNA extraction was performed using RNeasy^®^ Mini kit as described by the manufacturer (Qiagen, USA). Briefly, the tissues (not more than 30 mg) were disrupted and homogenized using a mortar and pestle followed by the addition of Buffer RLT to lyse the cells. The homogenized lysate was added with 70% ethanol and transferred to RNeasy spin column before centrifugation for 15 seconds at 8000 *g*. The flowthrough in the collection tube was mixed well with 70% ethanol and transferred to an RNeasy spin column placed in a new collection tube, followed by centrifugation for 15 seconds at 8000 *g.* The flowthrough was discarded. Similar steps were repeated using buffers RW1 and RPE. To ensure that there was no excess ethanol during the RNA elution, RNeasy spin column was centrifuged for 2 minutes at 8000 *g* after the addition of a second Buffer RPE. Finally, RNase-free water was directly added to the RNeasy spin column membrane and centrifuged for 1 minute at 8000 *g* to elute the RNA.

The integrity of RNA was confirmed by agarose gel electrophoresis. RNA concentration and purity were quantified using spectrophotometry by measuring the absorbance at 260 nm and 280 nm (A260/A280) and 260 nm and 230 nm (A260/A230). The range value of approximately 1.8 to 2.0 is optimal for RNA purity. Intact mammalian total RNA demonstrated sharp and clear 28S band and 18S band upon gel electrophoresis. All the samples were standardized as 1 µg/µl. Finally, RNA samples were stored at –80ºC for up to 6 months.

**cDNA synthesis**

The same amount of total RNA (5 μg) from each sample was reverse transcribed to cDNA in a final volume of 20 μl using RT^2^ First strand kit. The genomic DNA elimination mix was prepared (Table A) and mixed for each sample before incubation at 42^o^C for 5 minutes and was placed immediately on ice for 1 minute. The samples were added with a reverse-transcription mix (Table B), mixed and incubated at 42^o^C for 15 minutes. The reaction was later immediately stopped by further incubation at 95^o^C for 5 minutes. The cDNA samples were then diluted by adding 91 µl RNase-free water and finally stored at -20°C until used.

**Agarose gel electrophoresis**

Agarose gel 1.0% (w/v) was prepared by dissolving 0.4 g of agarose powder with 40 ml of 1× LB buffer. The mixture was heated in a microwave for 1 minute. The agarose was cooled under a running tap water. The melted agarose was further added with 3 µl of SYBR^®^ safe for staining and was mixed well before pouring into the casting tray which was already assembled with a comb. The gel was allowed to solidify at room temperature before placing into an electrophoresis tank filled with 1× LB buffer. For every sample, 1 µl of loading buffer was added to 5 µl of sample and was mixed well before loading into each well. DNA marker (1 kb) was assigned to the first lane. Electrophoresis was conducted at 90 V for 50 minutes. Bio-Rad gel doc imaging system was used to visualize and capture the bands.

**Table A.** Brief procedures of genomic DNA elimination mix RT^2^ first strand kit

| **Component** | **Volume / reaction (µL)** |
| --- | --- |
|  | **Sample per reaction** (1×) |
| RNA | 5.0 ( 5 µg) |
| Buffer GE | 2.0 |
| RNase-free water | 3.0 |
| Total per reaction | 10.0 |

**Table B.** Brief procedures of reverse-transcription mix RT^2^ first strand kit (Qiagen, USA)

| **Component** | **Volume / reaction (µL)** |
| --- | --- |
|  | **Sample per reaction** (1×) |
| 2× Buffer BC3 | 4.0 |
| Control P2 | 1.0 |
| RE3 Reverse Transcriptase mix | 2.0 |
| RNase-free water | 3.0 |
| Total per reaction | 10.0 |

**RT^2^ Profiler PCR array (Rat allergy and asthma pathway-related genes)**

The RT² Profiler PCR Array is a SYBR Green-optimized 96-well plate assay designed to investigate a panel of specific allergy and asthma pathway-related genes. To perform PCR array, 5 ng total RNA was subjected to real-time PCR cycle, with enzyme activation at 95°C for 10 minute, 40 cycles of denaturation at 95°C for 15 seconds, 60°C for annealing and extension for 1 minute each.

The C_T_ (comparative) method was used for data analysis and each gene fold-change was calculated as the difference in gene expression of the gene of interest and the reference house-keeping genes [β-actin (Actb), beta-2 microglobulin (B2m), hypoxanthine phosphoribosyltransferase 1 (Hprt1), lactate dehydrogenase A (Ldha) and ribosomal protein large P1 (Rplp1)], followed by delta-delta CT calculations [delta CT (Test Group)-delta CT (Control Group)]. Data analysis was performed using the manufacturer’s PCR array data analysis web portal.

**House dust mite–induced mouse model of asthma**

Female Balb/c mice were randomised into six different groups. (1) normal control (2) HDM, sensitised and challenged with HDM (3) LRE125, sensitised and challenged with HDM; treated with oral LRE (125 mg/kg) (4) LRE250, sensitised and challenged with HDM; treated with oral LRE (250 mg/kg) (5) LRE500, sensitised and challenged with HDM; treated with oral LRE (500 mg/kg) (6) Dex, sensitised and challenged with HDM; treated with i.p dexamethasone (3 mg/kg).

Prior to the animal work, HDM was dissolved and prepared to a concentration of 5000 μg/ml in PBS, and then was aliquoted and frozen at -20^o^C until use. In this study, 100 µg of whole body HDM extract in 20 μL phosphate-buffered saline (PBS) was introduced into female Balb/c mice intranasally on day 0, and subsequently exposed to 10 μg of HDM in 20 μL PBS on days 7, 8, 9, 10 and 11, followed by measurement of AHR on day 14 (adapted from Gregory *et al.* (2009) with modifications. Intranasal administration was conducted by initially bringing HDM aliquots (5 μg/μL) to room temperature and vortexed for 5 seconds at maximal speed. An anaesthetic cocktail containing ketamine and xylazine was prepared and administered at a dose of 50 mg/kg ketamine and 3.3 mg/kg xylazine (i.p.). The i.p administration was carried out gently by grabbing the dorsum of the mouse, with its head up and its rear legs down, while its tail was fixed between the little and ring finger. The anaesthetic solution was administered into the lower left quadrant of the peritoneum (5 μL/g).

The mouse was placed in the cage until no more vibrissae movement observed. Following anaesthesia, the mouse was held in a vertical position with its head up and its rear legs down, then 10 μL of HDM solution (at concentration that correspond to intended amount of HDM to be administered for sensitisation or challenge) was administered to each nostril dropwise using sterile tips (20 μL/mouse). The mouse was maintained in vertical position for at least 1 min to allow full distribution of the solution into the airways. The mouse was then placed in horizontal decubitus plane within the cage until the mouse returned completely to consciousness. LRE treatments (125, 250 and 500 mg/kg) were given orally one hour prior to challenge. On day 14, the mice were anesthetised using 100 mg/kg ketamine and 10 mg/kg xylazine and tracheotomy was performed (Figure B).

HDM sensitisation (100 µg i.n)

HDM challenged (10 µg i.n)

AHR

0

Day

7

8

9

10

11

14

Figure B. HDM-asthma induction protocol. Animals were intranasally (i.n) sensitised using 100 µg of HDM on day 0, this was followed by daily HDM intranasal challenged (10 µg) on day 7-11. Samples were collected for analysis on day 14, while animals that were scheduled for AHR test proceeded with their respective test.

**Measurement of airway hyperresponsiveness (AHR)**

AHR was measured on Day 14. Mice were anaesthetised by an i.p injection using 100 mg/kg ketamine and 10 mg/kg xylazine. A tracheotomy was then performed and trachea was cannulated with a tube with blunt end (20G) for connection to the nebulizer for administration of 1x PBS and methacholine (Sigma-Aldrich). Each mouse was placed in a whole-body plethysmograph chamber and ventilated mechanically by a ventilator at a tidal volume of 200 µl/breath and a breathing rate 150/min. Airflow and pressure changes were detected by respective transducers, recorder and analysed by FinePointe™ RC System (Buxco Research Systems, New Brighton, MN, USA) as described by Goh *et al.,* (2012). Airway resistance (RI) was measured in response to increasing concentrations of methacholine (Mch) (1, 2, 4, 8, 16 and 32 mg/ml). PBS (10 µl) and Mch (10 µl) was added to the nebulizer at 4 min intervals continuously. Results were expressed as a percentage of the respective basal values in response to 1x PBS.
