# Supplementary material for: Lignosus rhinocerotis Cooke Ryvarden ameliorates airway inflammation, mucus hypersecretion and airway hyperresponsiveness in a murine model of asthma
Source: PLoS One. 2021 Mar 30;16(3):e0249091. doi: 10.1371/journal.pone.0249091 (PMC8009377; doi:10.1371/journal.pone.0249091)
Supplement: Attachment — Submitted filename: Comments.pdf [file pone.0249091.s003.pdf]

Reviewer's Comment

The objective of the study is to assess the effect of Lignosus rhinocerotis Cooke Ryvarden on airway inflammation, mucus hypersecretion and airway hyper-responsiveness in a murine model of asthma. The manuscript is generally well written and the results are presented in good manner. The data supports the statements in results and discussion. However authors need to address the following.

1) The authors need to discuss the selection of the doses of LRE extract used in the present study.

2) Also need to provide the proper rationale for OVA induced asthma, preferably in the introduction.

3) More recent citations about inflammatory responses and airway hyper responsiveness to be added in the introduction.
