# Supplementary material for: Lignosus rhinocerotis Cooke Ryvarden ameliorates airway inflammation, mucus hypersecretion and airway hyperresponsiveness in a murine model of asthma
Source: PLoS One. 2021 Mar 30;16(3):e0249091. doi: 10.1371/journal.pone.0249091 (PMC8009377; doi:10.1371/journal.pone.0249091)
Supplement: Attachment — Submitted filename: Response to reviewer.docx [file pone.0249091.s004.docx]

Journal Requirements:

When submitting your revision, we need you to address these additional requirements.

 1. Please ensure that your manuscript meets PLOS ONE's style requirements, including those for file naming. The PLOS ONE style templates can be found at

https://journals.plos.org/plosone/s/file?id=wjVg/PLOSOne_formatting_sample_main_body.pdf and

https://journals.plos.org/plosone/s/file?id=ba62/PLOSOne_formatting_sample_title_authors_affiliations.pdf

*- Amended as required.*

2. As part of your revision, please complete and submit a copy of the Full ARRIVE 2.0 Guidelines checklist, a document that aims to improve experimental reporting and reproducibility of animal studies for purposes of post-publication data analysis and reproducibility: https://arriveguidelines.org/sites/arrive/files/Author%20Checklist%20-%20Full.pdf (PDF). Please include your completed checklist as a Supporting Information file. Note that if your paper is accepted for publication, this checklist will be published as part of your article.
*- Included on the submission as requested.*

Reviewers' comments:

Reviewer #1:

1) The authors need to discuss the selection of the doses of LRE extract used in the present study.

*During allergen stimulation, dendritic cells induce the differentiation of T cells into Th2 cells. Th2 cells secrete cytokines i.e. IL-4, IL-5 and IL-13 which facilitate recruitment and activation of eosinophils in the airway. Meanwhile Th2 cells will induce the production of IgE by the B cells via IL-4 and IL-13 stimulation.* Inflammatory mediators such as histamine and leukotrienes released by the eosinophils, T cells, macrophages, and neutrophils in the airway environment will result in damage to the airway, bronchoconstriction, and finally remodelling of the lung. In this study, oral administration of LRE at different doses (125, 250 and 500 mg/kg) showed varying responses. Among the doses tested in this study, LRE at 500 mg/kg significantly attenuated the level of IgE in serum, Th2 cytokines IL-4, IL-5 and IL-13 in BALF as well as leukocyte infiltrations in the lung tissues, in contrast to the other two lower doses, 125 and 20 mg/kg which were not consistently attenuated the responses. Based on the dose-response study, LRE 500 mg/kg was chosen for the subsequent studies.

2) Also need to provide the proper rationale for OVA induced asthma, preferably in the introduction.

*Sensitisation methods utilising ovalbumin (OVA) and house dust mite (HDM) are known to enhance manifestations of asthmatic features. OVA- and HDM-induced model are considered the appropriate methods for experimental allergic asthma. These models have similar clinical symptoms to human asthma, which are characterized by airway mucosal oedema, bronchial wall thicknesses, mucus hypersecretion and increased infiltration of inflammatory cells into the lung. However, there has been limited success with OVA-induced model and only moderate pulmonary inflammation and mild AHR have been observed. HDM has become more commonly used in mouse models to induce AHR because it has immunogenic properties, so the use of an adjuvant is not required. In addition, inhaled delivery of HDM has been more successful in inducing AHR, possibly because of the intrinsic enzymatic activity of this allergen.*

3) More recent citations about inflammatory responses and airway hyper responsiveness to be added in the introduction.

*During allergen stimulation, dendritic cells induce the differentiation of T cells into Th2 cells. Th2 cells secrete cytokines i.e. interleukin (IL)-4, IL-5, IL-9 and IL-13 which facilitate recruitment and activation of eosinophils in the airway. IL-5 and IL-9 are critical for promoting tissue eosinophilia and mast cell hyperplasia, whereas IL-13 stimulates mucus production by goblet cells and airway hyperresponsiveness (AHR). Meanwhile, Th2 cells will induce the production of IgE by the B cells via IL-4 stimulation. In the presence of airway inflammation, the cellular components especially the eosinophils and mast cells will be prompted to release a number of different mediators with the capacity to cause AHR. The i*nflammatory mediators such as IL-13, histamine, major basic proteins (MBP) and leukotrienes are known to cause AHR which lead to bronchoconstriction, and finally remodelling of the lung.

Reviewer #2:

1. This study is descriptive without any intervention design. The authors are encouraged to include more discussion points along possible mechanism.

*Added as suggested. Please see in discussion section.*

2. The dose effect of LRE is required in the study.

*Some preliminary works to determine dose-response effect are included.*

***Effects of LRE on IgE production in serum***

*As shown in Fig. 1, the OVA-induced asthmatic rats (OVA group) presented significantly increased IgE levels compared to normal group. Significant decreased in the mean serum of IgE level was observed after treatments with LRE at 250 and 500 mg/kg (p < 0.05) when compared with OVA group. However, no significant decrease could be observed from the rats treated with LRE 125 mg/kg (Fig. 1).*

***Effects of LRE on Th2 cytokines in BALF***

*As shown in Fig. 2, the levels of IL-4, IL-5 and IL-13 were significantly increased in BALF of OVA group. Treatment with LRE 500 mg/kg resulted in significantly decreased of IL-4, IL-5 and IL-13 levels when compared to OVA group. In contrast, treatment with LRE 250 mg/kg significantly decreased only IL-5 level, while LRE 125 mg/kg significantly decreased IL-4 and IL-5 levels.*

***Effects of LRE on histopathological change in lung tissue***

*Histopathological analysis by H&E staining demonstrated that lung tissue exposed with OVA had a marked increase in the leukocyte infiltration into the lung tissues. Treatment with LRE 250 or LRE 500 mg/kg significantly decreased infiltration of leukocytes in the peribronchial region and perivascular connective tissue of the airway compared with that observed in the OVA group (Fig. 3). No significant attenuation was observed by the LRE 125 mg/kg treatment group. Based on the dose-response study, LRE at the 500 mg/kg concentration was selected for the downstream experiments.*

3. What is the difference between those two allergic models? The rationale for the LRE applied to those similar model is not clear.

*Some points on OVA and HDM models are included in the introduction.*

*This study was started using ovalbumin-induced model. Using this model, we were able to induce a significant level of inflammation characterised by elevated IgE level, Th2 cytokines level, leukocytes infiltration and mucous in the lung tissue. However, when we tested AHR using OVA-induced model, we were not able to induce significant AHR level in the mouse model. Therefore, for AHR study, we used house dust mite (HDM) to induce the mice. The result as presented in this manuscript.*

4. In ANOVA, the dependent variable must be a continuous. The statistic employed in the mucus score not appropriate.

*Data were expressed as mean ± standard deviation (SD). Statistical differences were assessed by one-way analysis of variance (ANOVA) test followed by Scheffe’s post hoc correction and Student’s t test using Statistical Programme for Social Science (SPSS) version 20.0 (New York, USA).* p *< 0.05 was considered statistically significant.*

5. What is the index for stability of the pharmaceutical product?

*The product shelf life is 5 years.*
