# Supplementary material for: Lignosus rhinocerotis Cooke Ryvarden ameliorates airway inflammation, mucus hypersecretion and airway hyperresponsiveness in a murine model of asthma
Source: PLoS One. 2021 Mar 30;16(3):e0249091. doi: 10.1371/journal.pone.0249091 (PMC8009377; doi:10.1371/journal.pone.0249091)
Supplement: Attachment — Submitted filename: Rebuttal letter 100321.docx [file pone.0249091.s005.docx]

**Response to reviewer:**

*6. Review Comments to the Author*

Please use the space provided to explain your answers to the questions above. You may also include additional comments for the author, including concerns about dual publication, research ethics, or publication ethics. (Please upload your review as an attachment if it exceeds 20,000 characters)

Reviewer #1: (No Response)

Reviewer #2: The author’s responses to common # 5 do not reach my expectations. Please choose the right statistical test for mucus score which is discontinuous data.

Thank you for the comment. For mucus score results, as the ordinal data are not normally distributed, Kruskal Wallis test is used as the appropriate test to compare the median among groups.  The correct statistical test is mentioned and rephrased.

**Statistical analysis**

 Data were expressed as mean ± standard deviation (SD). Statistical significance (p<0.05) was determined by a one-way ANOVA test followed by Scheffe’s post hoc correction using Statistical Programme for Social Science (SPSS) version 20.0 (New York, USA). For inflammation and mucus scoring, statistical analyses were performed using  Kruskal Wallis tests with confidence interval adjustment by Bonferroni correction. The data from PCR array studies were analysed by the web-based software (SABiosciences) using a 2−∆∆CT method. The expression level for genes of interest was normalized by the expression of housekeeping genes.
